# Disease activity and anti-topoisomerase I antibody positivity are associated with elevated serum B cell activating factor levels and a B cell subset-wide reduction of BAFF-receptor expression in SSc

**DOI:** 10.1093/rheumatology/keag354

**Published:** 2026-07-01

**Authors:** Abdulellah M Alzahrani, Stefan F H Neys, Wieke M van Oostveen, Saad Ahmed, Ew Nivine Levarht, Rudi W Hendriks, Odilia B J Corneth, Hans Ulrich Scherer, Rene E M Toes, Jeska K de Vries-bouwstra, Cynthia M Fehres

**Affiliations:** Department of Rheumatology, Leiden University Medical Center (LUMC), Leiden, The Netherlands; Department of Medical Laboratory Technology, King Abdulaziz University, Jeddah, Saudi Arabia; Department of Pulmonary Medicine, Erasmus MC, University Medical Center, Rotterdam, The Netherlands; Department of Rheumatology, Leiden University Medical Center (LUMC), Leiden, The Netherlands; Department of Rheumatology, Leiden University Medical Center (LUMC), Leiden, The Netherlands; Department of Rheumatology, Leiden University Medical Center (LUMC), Leiden, The Netherlands; Department of Pulmonary Medicine, Erasmus MC, University Medical Center, Rotterdam, The Netherlands; Department of Pulmonary Medicine, Erasmus MC, University Medical Center, Rotterdam, The Netherlands; Department of Rheumatology, Leiden University Medical Center (LUMC), Leiden, The Netherlands; Department of Rheumatology, Leiden University Medical Center (LUMC), Leiden, The Netherlands; Department of Rheumatology, Leiden University Medical Center (LUMC), Leiden, The Netherlands; Department of Rheumatology, Leiden University Medical Center (LUMC), Leiden, The Netherlands

**Keywords:** SSc, B cell activating factor, BAFF-receptor, disease activity, autoantibody subset

## Abstract

**Objectives:**

Although B cell activating factor (BAFF) serum levels are reported to correlate with the extent of skin fibrosis and markers of systemic inflammation in SSc, their relationship with BAFF-receptor (BAFF-R) expression levels and their potential as biomarker for active disease is not investigated so far. This study was undertaken to investigate BAFF/BAFF-R dynamics in SSc and how they relate to autoantibody subtype and disease activity.

**Methods:**

Peripheral blood mononuclear cells of 69 SSc patients (ACA+: *n* = 37; ATA+: *n* = 32) and 10 age- and sex-matched controls were stained for BAFF-R followed by flow cytometry analyses. Serum BAFF and autoantibody levels were measured by ELISA.

**Results:**

BAFF levels were significantly elevated in SSc patients compared with the controls. This was more pronounced in clinically active than clinically stable patients, and in ATA+ compared with the controls and ACA+ SSc patients. Concurrently, BAFF-R expression was reduced on total and on several B cell subsets. BAFF-R expression correlated inversely to serum BAFF levels. In addition, BAFF levels correlated with ESR and CRP in ATA+ SSc patients. Of note, we observed a moderate correlation between BAFF and ATA-IgG, but not between BAFF and ACA-IgG.

**Conclusions:**

Our study indicates that elevated BAFF levels, coupled with a reduction in BAFF-R expression, are significant characteristics in ATA+ SSc and of clinically active patients regardless of autoantibody status. Furthermore, BAFF levels show correlations with markers of systemic inflammation and autoantibody levels, specifically in ATA+ SSc. These findings might indicate distinct regulation of B cell responses in ATA+ and ACA+ SSc, particularly emphasizing their relevance in the context of clinically active SSc.

## Graphical abstract

**Figure:**
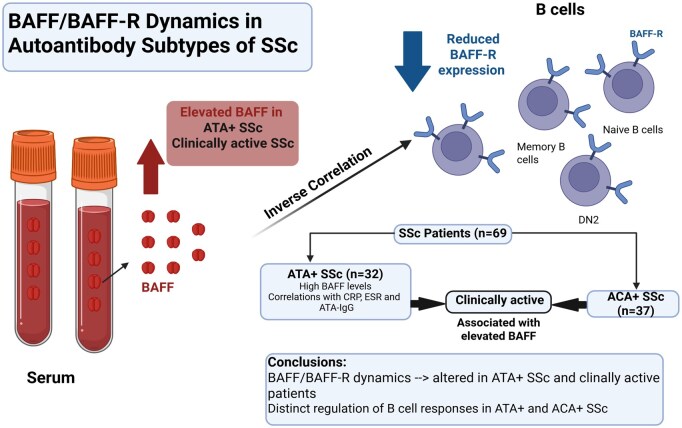

Rheumatology key messages

- Clinically active SSc patients exhibit higher BAFF levels, irrespective of autoantibody subgroup.
- Serum BAFF levels show an inverse relationship with BAFF-R expression on B cells.
- In ATA+ SSc, BAFF levels correlate with systemic inflammation markers (CRP and ESR) and autoantibody levels.

## Introduction

SSc is a rare multi-organ autoimmune disease characterized by vasculopathy, fibrosis of skin and internal organs, and immunologic abnormalities. Autoreactive B cell responses against nuclear antigens are found in >95% of the patients [1]. Several ANAs have been identified in SSc, including but not limited to anti-topisomerase-1 antibodies (ATAs), anti-centromere protein B antibodies (ACAs) and anti-RNA polymerase III antibodies, where ATA and ACA are the most prevalent and each is associated with clinically distinct subgroups of SSc [2]. ATA responses are associated with a poor prognosis, while ACA responses are associated with slower disease progression [3–6]. Furthermore, in SSc patients, the naïve B cell compartment in circulation is expanded, and the memory B cell compartment is diminished, but displays an active phenotype [7].

B cell activating factor (BAFF), also referred to as B lymphocyte stimulator BlyS, is part of the tumour necrosis factor (TNF) superfamily [8]. It is primarily recognized for its function in supporting B cell survival and maturation [9]. BAFF is secreted by a variety of immune cells, including neutrophils, monocytes, dendritic cells, macrophages and T cells [9, 10]. It binds to three known receptors from the TNF receptor superfamily: B cell maturation antigen (BCMA) (TNFRSF17), transmembrane activator and calcium modulator and cyclophilin ligand interactor (TACI) (TNFRSF13B) and BAFF-receptor (BAFF-R/BR3; TNFRSF13C) [11, 12]. Those receptors are mainly expressed on B cells [10, 13], with BAFF-R serving as the key receptor mediating BAFF’s biological effects [11].

Recent advancements in CD19 chimeric antigen receptor (CAR) T cell therapies have shown promise in treating SSc patients, demonstrating that targeting specific immune pathways can lead to significant clinical improvements [14, 15]. Moreover, it underscores the critical role of B cells in the pathogenesis of SSc. Therefore, a better understanding of the regulatory mechanisms governing B cells, potentially through pathways like BAFF/BAFF-R, may enhance therapeutic strategies and optimize patient outcomes in SSc.

The interactions between BAFF/BAFF-R are indispensable for B cell survival throughout all maturation and differentiation stages [12]. In mice, increased BAFF levels rescue autoreactive B cells from going to anergy, indicating a potential contribution of dysregulated BAFF levels in the development of autoimmunity [16]. In humans, elevated BAFF levels have been studied in individuals with SSc, SLE, RA and SjS [13, 17, 18]. In these conditions, increased BAFF levels are associated with higher levels of disease-specific autoantibodies, such as anti-double-stranded DNA, RF and anti-Ro/La antibodies [17, 19, 20]. Furthermore, studies have shown a significant positive correlation between serum BAFF levels and the extent of skin fibrosis in SSc [13, 21], suggesting a role for BAFF in altering B cell homeostasis and disease progression. In line with this, in tight skin (TSK/+) mice, serum BAFF levels were shown to be upregulated, and BAFF antagonists inhibited the development of skin fibrosis and autoantibody production. Nevertheless, in a clinical trial involving 20 patients with early active diffuse cutaneous SSc, administration of an anti-BAFF monoclonal antibody (Belimumab) did not result in more skin improvement as compared with placebo while a marked reduction in the expression of B cell signalling and profibrotic genes and pathways was observed, a pattern not seen in the placebo group [22]. Despite the reports on distinct serum BAFF levels in SSc, little is known about the regulation of BAFF-R expression in SSc [23].

In the current study, we have examined serum BAFF levels in a cohort of 69 SSc patients, of which 25/69 were classified as clinically active at the moment of sampling. Additionally, we have studied the expression of BAFF-R on total B cells as well as on different B cell subsets. We correlated BAFF levels to patients’ clinical parameters and autoantibody titres. Collectively, our results suggest that the BAFF/BAFF-R axis is implicated in aberrant B cell responses and a characteristic of active disease in SSc.

## Methods

### Patients and healthy individuals

In this cross-sectional observational study, peripheral blood was obtained from ACA+ SSc patients (*n* = 37) and ATA+ SSc patients (*n* = 32) visiting the outpatient clinic of the Department of Rheumatology at Leiden University Medical Center (LUMC). Additionally, age- and sex-matched healthy donors (HDs) (*n* = 10) were recruited from the LUMC Voluntary Donor Service (LUVDS). All patients fulfilled the ACR/EULAR 2013 criteria for the classification of SSc, did not receive B cell depleting medications and had not undergone haematopoietic stem cell transplantation in the past. Patients’ characteristics are outlined in Table 1. This study was approved by the ethical review board of the Leiden University Medical Center (protocol number P17.151) and the Leiden University Biobank Toetsing Commissie (REU/036/SH/sh), and written informed consents were obtained from both patients and HDs. Peripheral blood mononuclear cells (PBMCs) were obtained by Ficoll-Paque gradient centrifugation and frozen upon isolation.

**Table 1:** Patient characteristics at the time of peripheral blood collection.

	HD (*n* = 10)	Total SSc (*n* = 69)	**ACA+ (*n* = 37)** ^a^	ATA+ (*n* = 32)
Age (years)	58 ± 17.2	56 ± 11.2	58 ± 10.6	54 ± 11.7
Female sex, *n* (%)	8 (80)	49 (71)	29 (78.4)	20 (62.5)
RP (months)		158 (85–277)	226 (104–338)	119 (57–219)
Disease duration^b^ (months)		85 (45–166)	86 (48–166)	82 (29–149)
Disease subset				
Non cutaneous, *n* (%)		10 (14.7)	10 (27.8)	0 (0)
Limited cutaneous, *n* (%)		36 (52.9)	23 (63.9)	13 (40.6)
Diffuse cutaneous, *n* (%)		22 (32.4)	3 (8.3)	19 (59.4)
mRSS		3 (2–7)	2 (1–4)	5 (2–14)
ILD,^c^ *n* (%)		27 (39.1)	5 (13.9)	22 (68.8)
Renal crisis (ever), *n* (%)		2 (2.9)	0 (0)	2 (6.3)
Digital ulcers, *n* (%)		6 (8.8)	4 (11.4)	2 (6.3)
GAVE, *n* (%)		1 (1.5)	1 (2.8)	0 (0)
PAH, *n* (%)		3 (4.4)	2 (5.6)	1 (3.1)
CRP (mg/dl)		3 (3–4.5)	3 (2.4–4.5)	3.1 (3–4.5)
ESR (mm/h)		10 (6–31)	9.5 (6–29)	11 (6–37)
Use of any immunosuppressives,^d^ (%)		19 (27.5)	(16.7)	13 (40.6)

Parametric data are reported as mean ± S.D., non-parametric data as median (IQR).

a For clinical data, *n* = 36 for ACA+ patients; age and sex were available for all patients.

b Since first non-Raynaud symptom.

c ILD defined as on HRCT.

d Includes methotrexate, azathioprine, cyclophosphamide, mycophenolate mofetil, corticosteroids, hydroxychloroquine, biologicals.

ACA, anti-centromere protein B antibodies; ATA, anti-topoisomerase antibodies; HD, healthy donor; HRCT, high-resolution CT; ILD, interstitial lung disease; PAH, pulmonary arterial hypertension; GAVE, gastrointestinal involvement; IQR, interquartile range; mRSS, modified Rodnan skin score [x/51].

### Evaluation of patient’s disease activity

Two medical professionals independently evaluated disease activity in the studied patients. Disease activity was defined by the presence of active digital tip lesions, an increase in the modified Rodnan Skin Score (mRSS), a decline in pulmonary function, or new onset of pulmonary arterial hypertension (PAH), and/or renal crisis and/or interstitial lung disease (ILD), either at the time of assessment or within the preceding 3 months. Discrepancies between evaluators were resolved through discussion and reassessment.

### Flow cytometry

PBMCs were thawed and washed with PBS. Upon staining with BD Horizon Fixable Viability Stain 575V (BD Biosciences, cat. no. 565694, San Diego, CA), PBMCs were stained extracellularly with antibody mixes containing anti-CD3-AF700 (Invitrogen, lot# 1939050), anti-CD19-BV750 (Biolegend, lot# B346182), anti-CD27-BV421 (BD Biosciences, lot# 9273687), anti-CD21-BV605 (BD Biosciences, lot# 2045726), anti-CD86- BV650 (BD Biosciences, lot# 22097), anti-CD38-BV786 (Biolegend, lot# 2161663), anti-CD11c-BV480 (BD Biosciences, lot# 2038182), anti-BAFF-R-PerCP-Cy5.5 (Biolegend, lot# B318020), anti-IgD-BV711 (BD Biosciences, lot# 2168183), anti-IgG-PE-Cy7 (BD Biosciences, lot# 1271913), anti-IgM-APC-CY7 (BD Biosciences, lot# 1089387). Cells were not permeabilized and all markers were analysed as surface antigens. Samples were measured on a Symphony A5 flowcytometer (BD Biosciences). Data were analysed using BD FACSDiva™ (v9.0, BD Biosciences) and FlowJo software v10.9.0 (FlowJo , LLC, Ashland, OR).

### ATA and ACA ELISA

ACA and ATA isotype ELISAs (IgG, IgM and IgA) were performed on plasma of the patients and HDs. High-binding 384-well microplates (Corning) plates were coated with 1 μg/ml human CENPB in Citrate buffer pH 9.6 (Prospec; cat: pro-390-c) or with 0.5 μg/ml human TOP1 in PBS pH 8.0 (Prospec; cat: enz-306-b) incubated overnight at 4°C. Plates were washed next day with washing buffer (PBS/0.05% Tween) and blocked with (PBS/1% BSA) for IgG or (PBS/1%BSA/50 mM TRIS pH 8.0) for IgM and IgA. Samples and standards were diluted in (PBS/0.05% Tween/1% BSA) or with (PBS/0.05% Tween/1% BSA 50 mM TRIS) for ACA IgM and IgA, which then incubated for 60 min at 37°C. Plates were next incubated with the detection antibodies: Rabbit anti-human IgG-HRP (DAKO, Glostrup, Denmark; cat. P0214) diluted in (PBS/1%BSA/0.05%Tween), Goat anti-human IgA-HRP (Invitorgen, Thermo Fisher Scientific; cat. A18781, USA) and Goat anti-human IgM-HRP (Millipore, Germany; cat. AP114P). ELISAs were developed using ABTS/0.15% H_2_O_2_. Optical density was measured at 415 nm with a Multiskan™ FC Microplate Reader (Thermo Fisher Scientific, USA) using SkanIt™ Software (Thermo Fisher Scientific, USA).

### BAFF ELISA

Human BAFF/BLyS/TNFSF13B ELISA was performed according to the manufacturer R&D systems (cat# DY124-05). Briefly, plates were coated with capture antibodies (cat. DY124-05; R&D Systems, Bio-Techne, Minneapolis, MN) in a concentration of (1 μg/ml) and incubated overnight at room temperature (RT). The second day, plates were washed with washing buffer (0.05% Tween 20 in PBS) and then blocked with 1% BSA in PBS. Samples and standards were incubated for 2 h at RT. After that, detection antibodies were added in concentration of (10 ng/ml) for 2 h at RT. Multiple washing cycles followed before adding the Streptavidin-HRP for 20 min in the dark at RT. ELISAs were developed using ABTS/0.15% H_2_O_2_. Optical density was measured at 415 nm with a Multiskan™ FC Microplate Reader (Thermo Fisher Scientific, USA) using SkanIt™ Software (Thermo Fisher Scientific, USA).

### Statistical analysis

Statistical analysis was performed with GraphPad Prism software version 10.6.1 (GraphPad Software, Boston, MA). BAFF levels and BAFF-R expression were compared between patients and HD and between patient subgroups defined by autoantibody status and clinical disease activity using Kruskal–Wallis or Mann–Whitney tests for non-parametric values where appropriate. Non-parametric correlations between BAFF and clinical parameters were assessed using Spearman’s rank correlation. Clinical parameters were selected based on previous studies reporting associations between BAFF and skin fibrosis (mRSS) and markers of systemic inflammation such as ESR. *P* < 0.05 was considered as statistically significant.

## Results

### BAFF-R expression is significantly reduced in peripheral blood B cells from SSc patients

**Figure 1:**
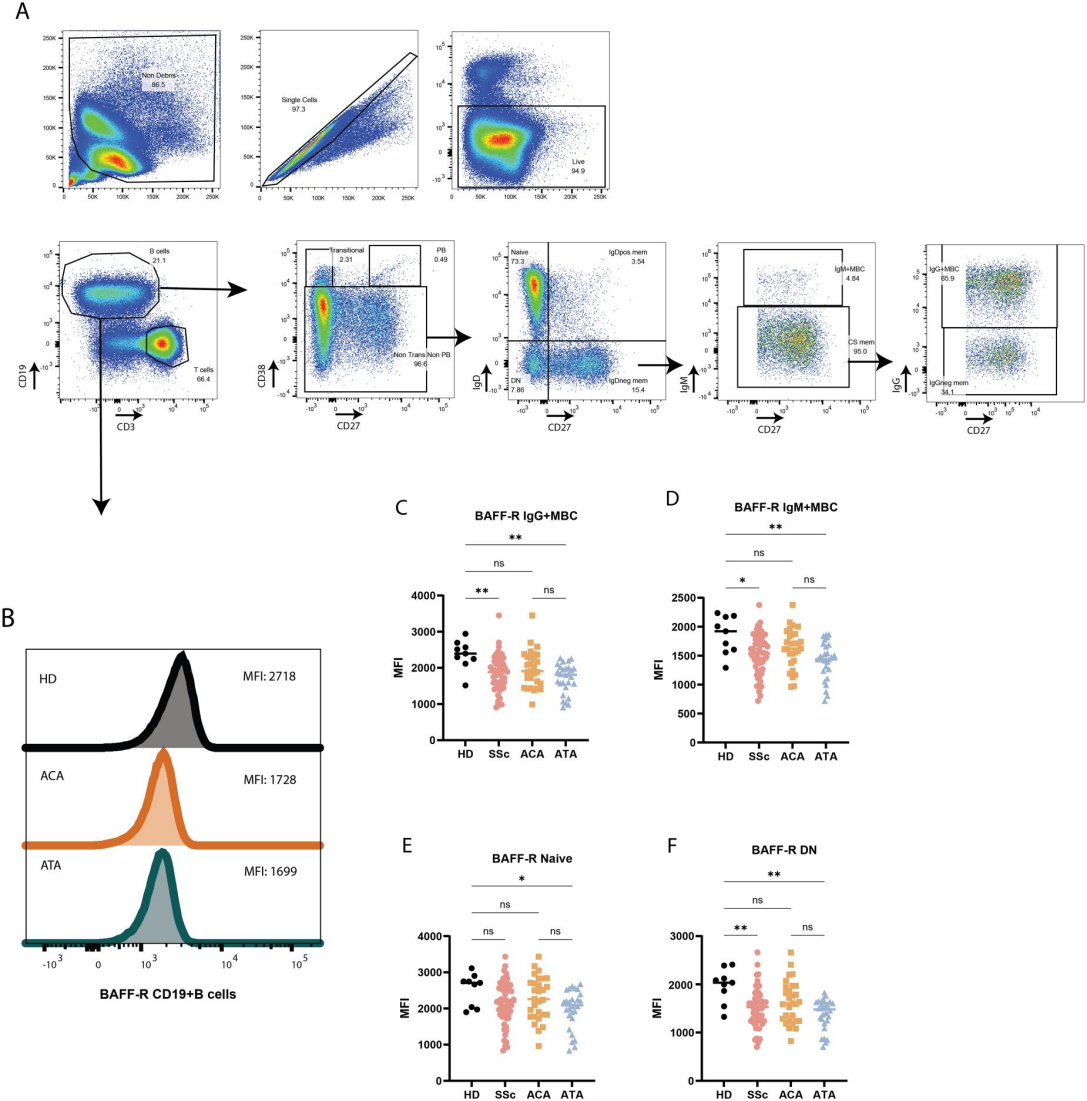
Expression of BAFF-receptor (BAFF-R) on different B cell subsets. (**A**) Gating strategy showing different B cell subsets, where four subsets were depicted as follows: IgG+ MBC, IgM+ MBC (within CD27+ compartment), Naïve B cells and double negative B cells (DN) (within CD27-compartment). (**B**) BAFF-receptor expression on CD19+ B cells shown in a representative histogram of HD, ATA+ SSc and ACA+ SSc patient. Expression was depicted as median fluorescence intensity (MFI). Group data are presented as mean ± S.D. of BAFF-R median MFI on CD19+ B cells: HD 2288 ± 335.8 (*n* = 9), ACA 2135 ± 556.5 (*n* = 29), ATA 1861 ± 471 (*n* = 28). (C–F) Expression of BAFF-R on different B cell subsets depicted as MFI. Comparisons were depicted between HD vs SSc as well as ATA+ SSc and ACA+ SSc. All *P*-values were calculated with Kruskal–Wallis test. ns = non-significant, * = *P* < 0.05, ** = *P* < 0.01

We assessed BAFF-R expression on B cell subsets of SSc and HD PBMCs. The gating strategy to specifically study the B cell subsets and the expression of BAFF-R is shown in Fig. 1A. B cells of ATA+ SSc patients and ACA+ SSc patients exhibited lower expression of BAFF-R compared with HD B cells as shown in the representative histograms (Fig. 1B). In addition, BAFF-R expression was significantly lower in B cell subsets of SSc patients, including IgG+ memory B cells (MBC) (C), IgM+ MBC (D), naïve B cells (E) and DN (F) compared with HD. Of note, these results were mainly found in ATA+ SSc, as there were no significant differences found when comparing BAFF-R expression on ACA+ B cells compared with HD B cells (Fig. 1C–F). These results show that BAFF-R expression is decreased on B cells of SSc patients, which is more pronounced in ATA+ SSc.

### BAFF-R expression on B cells of clinically active vs clinically stable SSc patients

**Figure 2:**
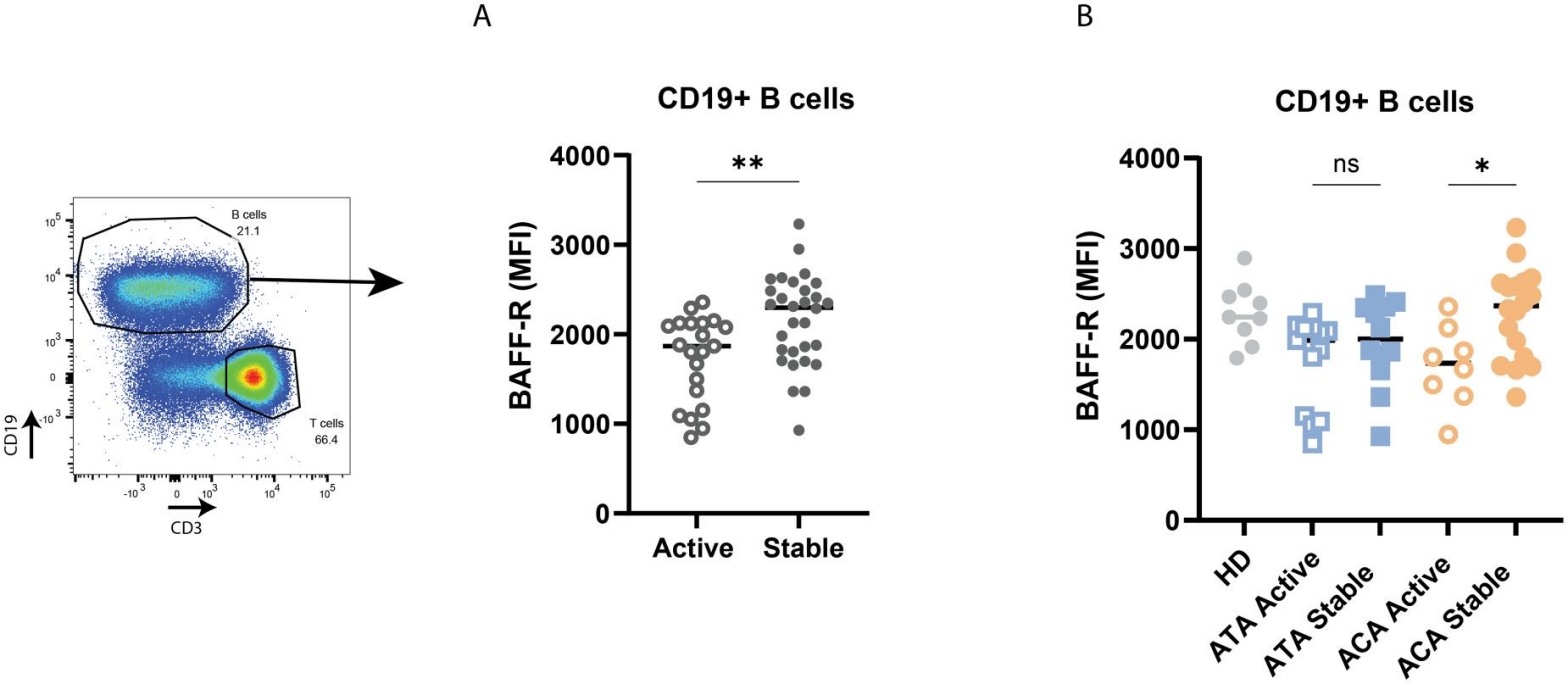
Expression of BAFF-receptor on SSc patients stratified on their serologic and disease status. (**A**) Expression of BAFF-R on B cells depicted as median fluorescence intensity among total active patients vs total clinically stable patients. (**B**) Expression of BAFF-R on total B cells depicted as median fluorescence intensity among active vs clinically stable ACA+ SSc and active vs clinically stable ATA+ SSc patients. All *P*-values were calculated with Kruskal–Wallis test. ns = non-significant, *P < 0.05, **P < 0.01

B cells of clinically active SSc patients showed significantly lower expression of BAFF-R compared with B cells of patients with stable disease (Fig. 2A). Of interest, after stratifying for autoantibody status, the data show that this difference is mainly present in the ACA+ SSc patients (Fig. 2B). This difference was not observed in B cells of ATA+ SSc patients with active disease. These results indicate that BAFF-R expression and BAFF levels might be differently regulated in SSc dependent on autoantibody subgroup.

### BAFF levels in serum of SSc patients correlate negatively with the expression of BAFF-R

**Figure 3:**
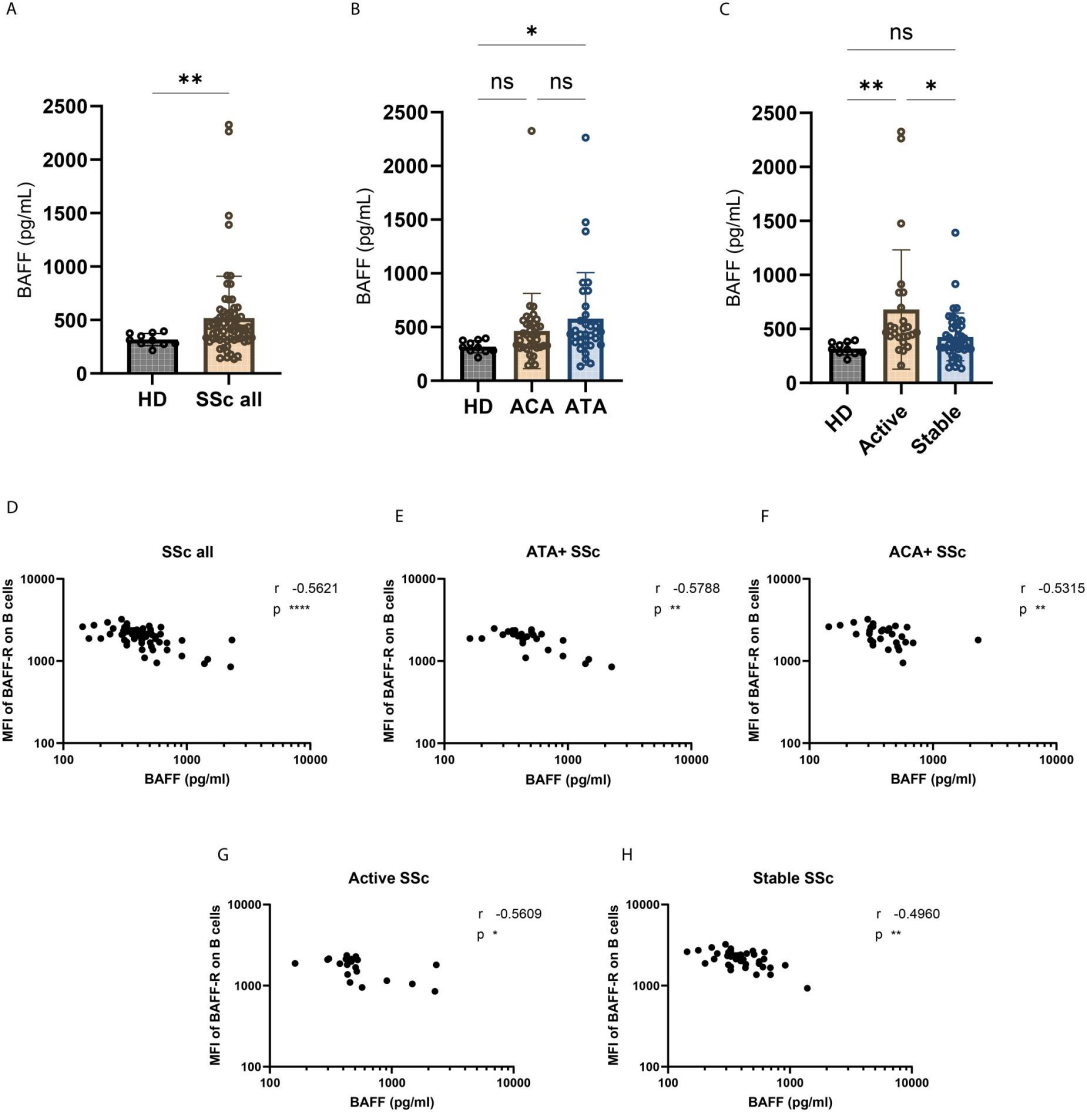
Serum BAFF levels assessed by ELISA are significantly upregulated in SSc patients compared with HD and inversely correlate with the expression of BAFF-R. (**A**) BAFF levels shown in pg/ml as assessed by ELISA on patients’ and HD serum. (**B**) BAFF levels shown in HD and in SSc patients stratified for autoantibody status. (**C**) Serum BAFF levels shown in HD, and SSc patients stratified for disease activity. (**D–H**) Correlation between BAFF levels in patients’ serum and the expression of BAFF-R on B cells of the same patients. Results also stratified based on the serological and disease status. All *P*-values were calculated with Kruskal–Wallis test and Mann–Whitney test, non-parametric correlations were assessed using Spearman’s rank correlation. ns = non-significant, *P < 0.05, **P < 0.01

**Figure 4:**
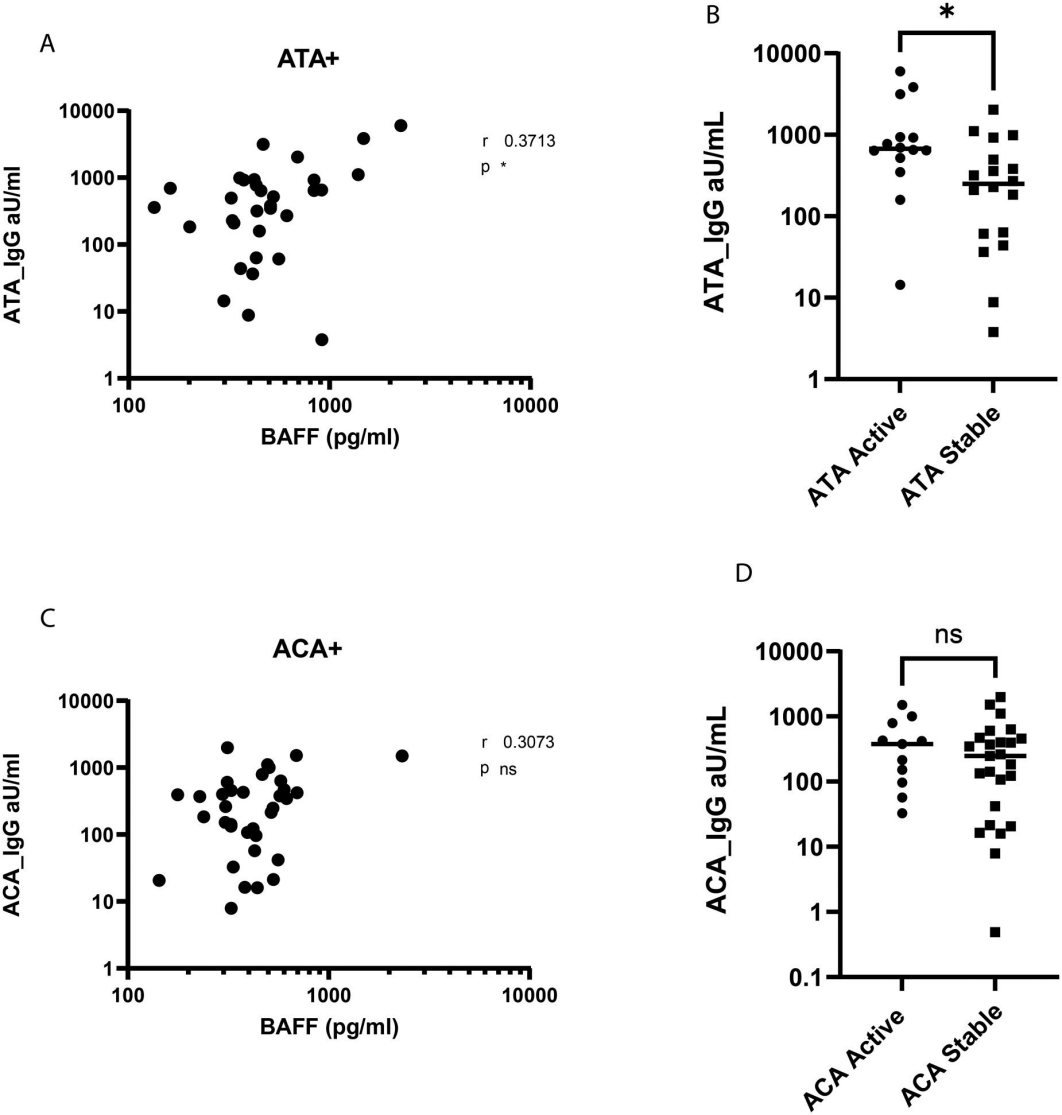
BAFF levels assessed by ELISA correlate with the titre of ATA-IgG autoantibodies in ATA+ SSc. (**A**) Correlation conducted between BAFF levels measured by ELISA on patients’ serum shown in pg/ml and the titre of ATA-IgG in ATA+ SSc. (**B**) ATA-IgG serum levels assessed by ELISA in aU/ml in both active and clinically stable ATA+ SSc patients. (**C**) Correlation conducted between BAFF levels measured by ELISA on patients’ serum shown in pg/ml and the titre of IgG autoantibody in ACA+ SSc. (**D**) ACA-IgG serum levels assessed by ELISA in aU/ml in both active and clinically stable disease SSc patients. *P*-values were calculated with Mann–Whitney test, non-parametric correlations were assessed using Spearman’s rank correlation. *P < 0.05, **P < 0.01

We next investigated BAFF levels in serum of SSc patients and HD. Overall, BAFF levels were significantly higher in SSc patients compared with HD (Fig. 3A). The elevation in BAFF was significant in ATA+ SSc, but not for ACA+ SSc (Fig. 3B). In addition, active SSc patients showed higher BAFF serum levels compared with clinically stable SSc patients and HD (Fig. 3C). Interestingly, serum BAFF levels correlated negatively with the expression of BAFF-R on total B cells of SSc patients (Fig. 3D). The significant negative correlation remained when stratifying for ATA+ SSc (Fig. 3E), ACA+ SSc (Fig. 3F) or active disease (Fig. 3G) as well as clinically stable disease (Fig. 3H). Furthermore, we observed a weak positive correlation between serum BAFF levels and the levels of ATA-IgG in ATA+ SSc (Fig. 4A). Interestingly, the ATA-IgG levels were significantly higher in active ATA+ SSc patients compared with clinically stable ATA+ SSc patients (Fig. 4B). No correlation was observed between serum BAFF levels and the levels of ACA-IgG in ACA+ SSc (Fig. 4C). in ACA+ SSc, active and clinically stable SSc patients showed similar ACA-IgG serum levels (Fig. 4D). Together, these data show that high BAFF serum levels coincide with low BAFF-R expression on B cells, which could be an indication of ongoing active B cell dysregulation that could be involved in driving autoantibody production in SSc.

### Weak correlations found between BAFF levels and patients’ ESR and CRP

**Figure 5:**
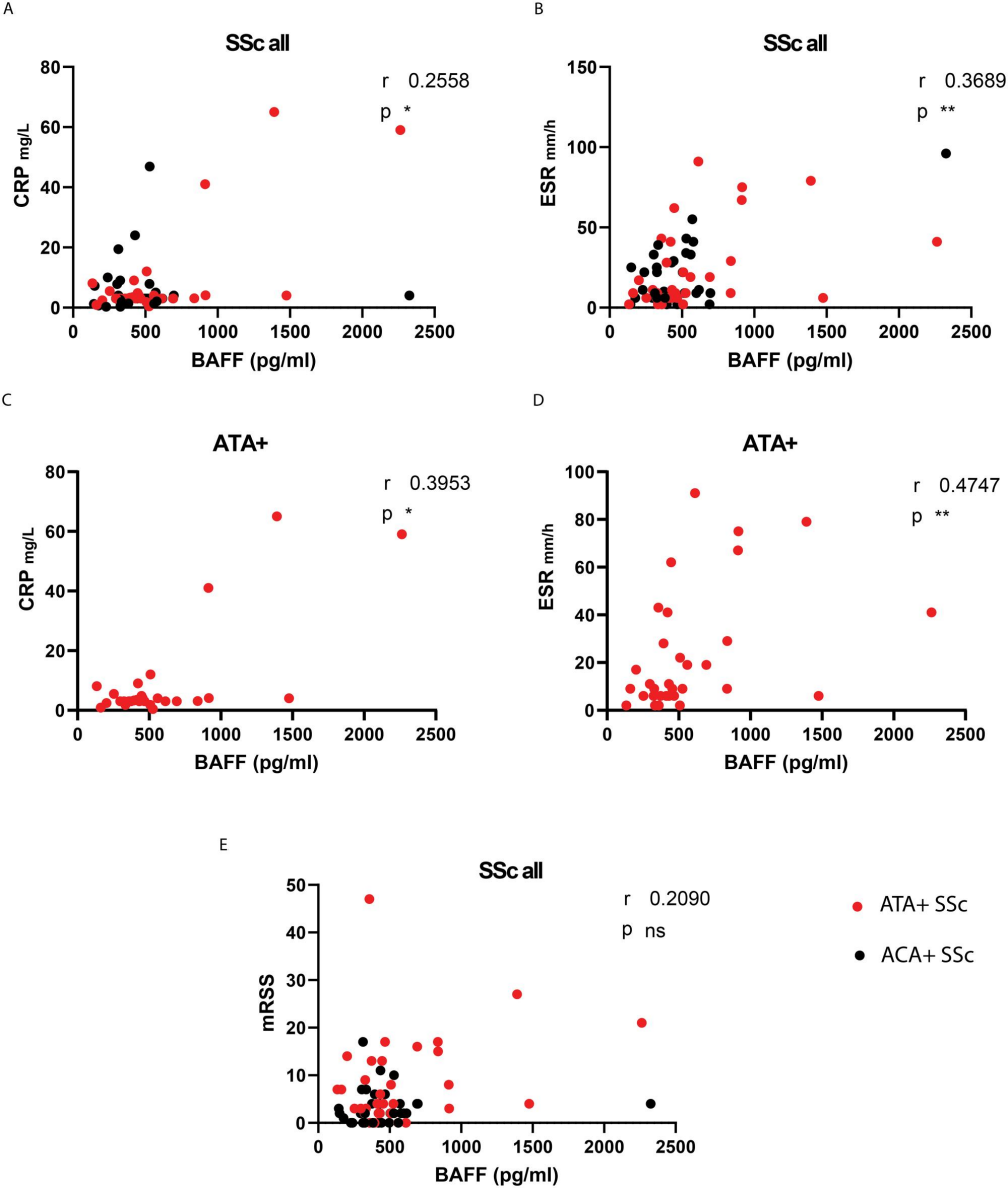
BAFF levels assessed by ELISA correlate with some of the patient’s clinical parameters. (**A**) BAFF levels measured by ELISA on patients’ serum shown in pg/ml correlates positively with CRP in total SSc. (**B**) BAFF levels shown in pg/ml correlates with ESR in total SSc. (**C–D**) BAFF levels measured by ELISA on patients’ serum shown in pg/ml correlates positively with CRP and ESR, respectively in ATA+ SSc. (**E**) BAFF levels shown in pg/ml correlates with modified Rodnan skin score (mRSS) in total SSc. Non-parametric correlations were assessed using Spearman’s rank correlation. ns = non-significant, *P < 0.05, **P < 0.01

Next, we assessed whether BAFF levels correlate with the clinical manifestations of SSc patients. To this end, we analysed possible correlations between BAFF and mRSS, presence of ILD, presence of digital ulcers and signs of systemic inflammation (measured by ESR and CRP). In the total SSc cohort, serum BAFF levels showed weak positive correlations with CRP and ESR, which aligns with previous findings (Fig. 5A and B) When the analysis was restricted to ATA+ SSc, the correlations between BAFF and both CRP and ESR were stronger (Fig. 5C and D), indicating that the correlations are largely driven by patients with ATA+ SSc phenotype. In line, BAFF‑R expression correlated negatively with CRP and ESR (Supplementary Fig. S1A and B). Notably, no correlation was detected between mRSS and serum BAFF levels when analysing the full cohort (Fig. 5E) nor when the analysis was restricted to the ATA+ or ACA+ SSc subgroups (data not shown). No significant associations were observed with the other clinical parameters evaluated in this cohort (mentioned above).

## Discussion

BAFF is crucial for B cell activation, differentiation and maturation. Serum BAFF levels have been reported to be elevated in SSc patients compared with HDs [13]. Here, we conducted a comprehensive study analysing BAFF-R expression on B cells, as well as serum BAFF levels in SSc. Our study shows an overall reduction of BAFF-R expression on B cells of SSc patients compared with HDs, which was most prominent in ATA+ SSc patients. This reduction of BAFF-R expression was seen in most of the B cell subsets analysed.

BAFF is known to be a potent factor supporting B cell survival [20]. In transgenic mice, overproduction of BAFF leads to elevation of serum antibodies reflecting a phenotype similar to that seen in SLE [20]. Aligning with previous reports, we observed a significant elevation of serum BAFF levels in SSc patients [13]. This elevation was highest when stratifying for ATA+ SSc. Notably, we observed significantly higher levels of BAFF in SSc patients with active disease compared with clinically stable disease. Our and previous studies have also shown that memory B cells from SSc patients display an activated phenotype [7]. Interestingly, BAFF was found to promote CD19 signalling and expression [24]. This might suggest that the enhanced B cell activity observed in SSc patients could be supported by elevated BAFF levels and subsequent enhancement of CD19 signalling.

We have stratified patients according to their disease status, based on the independent assessment of two clinicians. SSc patients with active disease show a stronger reduction in the expression of BAFF-R. It was previously shown that reduced BAFF-R expression associates with disease activity in SLE and SjS, as reduced BAFF-R was more pronounced in active SLE and SjS compared with healthy controls and patients with stable disease [25]. The data presented here show a similar pattern in SSc, with low BAFF-R expression being more pronounced in B cells from SSc patients with active disease. Furthermore, BAFF levels inversely correlated with the expression of BAFF-R on the B cell surface, which could hint towards the BAFF/BAFF-R axis being a biomarker for disease activity in SSs patients.

The increased BAFF levels observed in our cohort could potentially contribute to reduced BAFF‑R surface expression on B cells. In a BAFF‑transgenic mouse model, Lesley *et al.* [26] showed that sustained BAFF excess alters peripheral B‑cell selection by rescuing self-reactive B cells, supporting the concept that chronic BAFF exposure can reshape BAFF‑R-dependent B‑cell survival. Several mechanisms could explain the reduction of BAFF-R expression in SSc. Prolonged exposure to soluble BAFF has been shown in human B cells to cause downregulation of BAFF-R surface levels *in vitro*, even at BAFF concentrations similar to those observed in autoimmune disease’s patients, consistent with a direct BAFF-induced modulation of BAFF-R potentially via internalization or shedding [25]. Another mechanism that could potentially explains this phenomenon is through BAFF binding in the presence of TACI, which can trigger ADAM10/ADAM17-dependent cleavage of BAFF-R, leading to reduced surface receptor and altered BAFF-mediated survival signals [27, 28]. In parallel, transcriptional regulation of the BAFF-R promoter during B cell activation and differentiation can modulate BAFF-R expression at the mRNA level, providing an additional mechanism by which chronically stimulated B cell subsets might exhibit reduced BAFF-R phenotypes [29, 30]. Our *ex vivo* data though cannot distinguish between these mechanisms (ligand‑induced internalization, shedding or transcriptional down‑regulation), but they are compatible with a model in which chronic BAFF exposure in SSc contributes to BAFF‑R processing and reduced receptor availability on specific B‑cell subsets.

A previous study found that BAFF levels correlate with autoantibody titres in SjS [19]. Here, we described a positive correlation between BAFF and ATA-IgG serum levels in ATA+ SSc, which we consider is driven by the clinically active ATA+ SSc patients, as these patients showed significantly higher ATA-IgG serum levels compared with the clinically stable ATA+ SSc. We did not observe a significant correlation between BAFF and ACA-IgG serum levels. Whether the generally lower BAFF serum levels in ACA+ SSc are responsible for this, or whether the association is truly lacking, remains to be determined. The positive correlation between BAFF and ATA-IgG could indicate that BAFF might be directly involved in the regulation of autoantibody-producing plasmablasts/plasma cells in SSc.

BAFF is produced by a variety of cells including but not limited to: T cells, neutrophils and monocytes upon stimulation with pro-inflammatory cytokines including IFN-γ or G-CSF [25, 26, 31]. Previous studies have reported that higher levels of BAFF correlated with high autoantibody titres [19]. Since BAFF is known to be associated with promoting inflammation in other autoimmune diseases, we aimed to investigate whether BAFF levels correlated with the clinical parameters of patients. Thus, we investigated mRSS, as previous studies have shown a positive correlation between BAFF and mRSS [13, 21]. Our results revealed no correlation between BAFF and mRSS in the total cohort, nor when the analysis was restricted to ATA+ SSc or ACA+ SSc. Although this correlation has been previously reported by Sato *et al.* [13] and Shaker *et al.* [21], we speculate that this discrepancy is caused by the median mRSS reported in the studies. The median mRSS in our study cohort is significantly lower than the median mRSS studied in both studies [13, 21], possibly explaining why we do not find a correlation between BAFF levels and mRSS in our cohort. This is at least partially explained by the fact that 46% of the cohort in this study comprises ACA+ patients, which are characterized by limited skin involvement. Intriguingly, our data indicate that elevated BAFF levels are not limited to ATA+ SSc—typically characterized by a more inflammatory, clinically active and progressively fibrotic phenotype and therefore preferentially selected for anti-inflammatory clinical trials—but are also present in ACA+ patients with active disease. This finding reveals a previously underrecognized role for B cell dysregulation in ACA+ SSc, a subset more commonly viewed as a predominantly vascular, slowly progressive phenotype and frequently excluded from immunomodulatory trials. Our results therefore support reconsideration of this paradigm and argue for the inclusion of ACA+ patients in future BAFF-targeted therapeutic studies. Moreover, including dysregulation of the BAFF/BAFF-R axis as an inclusion criterion may help to identify a specific subgroup of SSc patients in whom anti-BAFF treatment (Belimumab) is most effective [22].

In addition to patient selection, it is important to consider how B cell-directed therapies themselves modulate the BAFF/BAFF-R axis. Given the central role of BAFF/BAFF‑R in B‑cell survival, it is relevant to consider how currently used B‑cell‑targeted therapies, including anti‑CD20 (rituximab) and belimumab, influence this axis. After rituximab therapy in several autoimmune diseases, serum BAFF levels rise markedly as a consequence of profound B‑cell depletion [32, 33]. In pemphigus and RA autoimmune diseases, decreased BAFF‑R expression on repopulating B cells after rituximab has been proposed to influence the kinetics of memory B‑cell reconstitution and the occurrence of disease relapses [34–36]. Conversely, BAFF inhibition with belimumab in SjS induces dynamic changes in BAFF‑R on CD27+ memory B cells, with an early increase followed by a decline in BAFF‑R intensity in parallel with a reduction in CD27+ B‑cell numbers over 6–12 months [37]. These observations indicate that both B‑cell depletion and BAFF neutralization profoundly affect serum BAFF levels and BAFF‑R expression, but there are currently no data in SSc patients on BAFF or BAFF-R expression after rituximab or belimumab, and therefore no evidence that these approaches restore BAFF levels in serum or BAFF-R expression on B cell subsets in SSc. Whether targeting the BAFF/BAFF‑R axis can normalize the reduction in BAFF‑R we observe in SSc thus remains an open question that will require longitudinal studies with detailed B‑cell phenotyping.

Interestingly, weak correlations were found with the inflammation marker ESR, aligning with what has been found in the report of Sato *et al.* [13]. In addition, we observed a weak correlation between BAFF and CRP. Although this relationship has not been investigated in SSc yet, BAFF levels correlated significantly to CRP in RA [38]. It is speculated that high BAFF levels could stimulate immune cells through binding to BAFF-R and subsequent activation of NF-κB pathways, resulting in augmented B cell and autoreactive B cell survival and activation. This, in turn, could subsequently promote autoantibody production, leading to formation of immune complexes that induces CRP and ESR [38].

In conclusion, this study provides novel evidence in support of the concept that the BAFF/BAFF-R axis is implicated in both ATA+ and ACA+ SSc. In ATA+ SSc, elevated BAFF levels are closely associated with systemic inflammatory markers and circulating autoantibody titres and are accompanied by reduced BAFF-R expression, consistent with a dysregulated B cell signalling environment that might directly contribute to disease activity. Notably, a similar BAFF/BAFF-R signature is also observed in patients with clinically active disease irrespective of autoantibody status, supporting a broader role for this pathway in dysregulated B cell activation in SSc. At the same time, our data reveal important differences between ATA+ and ACA+ patients, underscoring that while BAFF-driven mechanisms are shared, B cell regulatory pathways are differentially modulated across SSc subsets. Together, these findings highlight previously underrecognized B cell involvement in ACA+ SSc and refine current concepts of immune heterogeneity in SSc pathogenesis.

## Supplementary Material

keag354_Supplementary_Data

## Data Availability

The data that support the findings of this study are available on request from the corresponding author (c.m.fehres@lumc.nl).
