# Supplementary material for: Disease activity and anti-topoisomerase I antibody positivity are associated with elevated serum B cell activating factor levels and a B cell subset-wide reduction of BAFF-receptor expression in SSc
Source: Rheumatology (Oxford). 2026 Jul 1;65(7):keag354. doi: 10.1093/rheumatology/keag354 (PMC13415454; doi:10.1093/rheumatology/keag354)
Supplement: keag354_Supplementary_Data [file keag354_supplementary_data.docx]

**Supplementary figure and legend**

**Supplementary Figure S1. BAFF‑R expression on B cells correlates negatively with clinical inflammatory parameters CRP and ESR.**

**
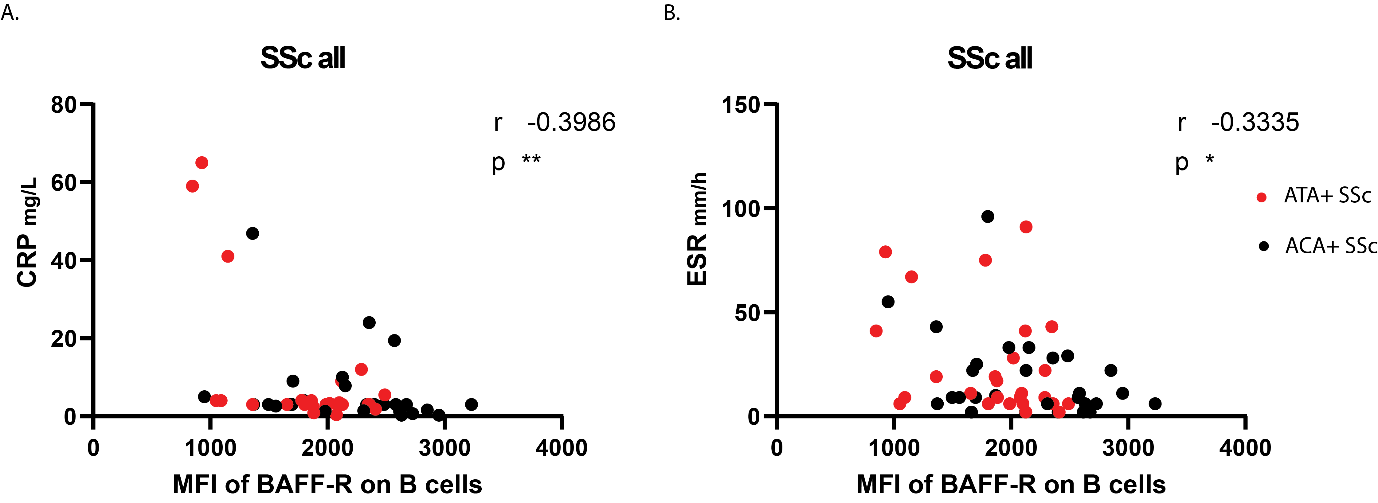
**

(A) BAFF‑R, depicted as median fluorescence intensity (MFI) on total B cells, correlates negatively with C‑reactive protein (CRP) in the total SSc patients. (B) BAFF‑R, depicted as MFI on total B cells, correlates negatively with erythrocyte sedimentation rate (ESR) in the total SSc patients. Non‑parametric correlations were assessed using Spearman’s rank correlation. ns, not significant; *p < 0.05; **p < 0.01.

**ALT TEXT:** Correlations plots showing a negative association between B cell activating factor receptor (BAFF‑R) expression on B cells in the entire systemic sclerosis (SSc) cohort and C-reactive protein (CRP) and erythrocyte sedimentation rate (ESR).
